# Relationship between Dietary Beef, Fat, and Pork and Alcoholic Cirrhosis

**DOI:** 10.3390/ijerph6092417

**Published:** 2009-09-10

**Authors:** Francis Stephen Bridges

**Affiliations:** Department of Health, Leisure and Exercise Science, The University of West Florida, 11000 University Parkway, Pensacola, FL 32514–5750, USA; E-Mail:fbridges@uwf.edu; Tel.: +1-850-474-2051; Fax: +1-850-474-2106

**Keywords:** liver cirrhosis, alcoholic, alcohol consumption, dietary fats, meat

## Abstract

Nanji and French [1] investigated the relationship between per-caput consumption of total fat, beef, and pork and for alcohol consumption and rates of mortality for cirrhosis for 16 countries for 1965. The present study reports significant and positive associations for 1996 and 2003 between the following: alcohol consumption and cirrhosis mortality, pork consumption and cirrhosis mortality, the product of alcohol and pork consumption and the product of alcohol and fat consumption. These supportive associations may represent a relationship between the risk of alcoholic cirrhosis and some heretofore unknown dietary or environmental factor related to conditions of pork or fat consumption. Limitations of the study design are discussed.

## 1. Introduction

Researchers investigated the per-caput consumption of total (dietary) fat, beef, and pork as dietary factors that might be, in addition to ethanol, related with mortality rates for cirrhosis in 16 countries for 1965, 11 European countries for the mid-70’s, and in ten Canadian provinces in 1978 [1]. They reported significant correlations between alcohol consumption and cirrhosis mortality and between pork consumption and cirrhosis mortality. They also reported a significant correlation between the product of both alcohol and pork consumption and rates of cirrhosis mortality. In countries with low alcohol consumption, *i.e.*, 5–10 l/caput/yr and 7.5–11.0 l/caput/yr, no correlations were obtained between alcohol consumption and rates of cirrhosis mortality. However, in these same countries significant correlations were obtained between pork consumption and rates of cirrhosis mortality.

The relative effect of alcohol and dietary factors in the pathogenesis of cirrhosis continues to be unclear. Some researchers [2] have reported a significant increase in the consumption of total pig products, pork, and offal in patients with three stages of alcoholic liver disease compared with controls. Still others [3] reported steatosis to be associated with cirrhosis. Fat as one dietary factor has been shown to influence the degree of steatosis consequent to alcohol abuse [1]. On the other hand, higher protein intake may protect against the development of alcoholic cirrhosis [4]. The fat content of beef and pork is similar; however, pork has more linoleic acid than beef [5]. Interestingly, others [6) postulated that linoleic acid facilitates development of alcoholic liver disease. These findings provide an explanation for their previous epidemiological observations in 1985.

## 2. Method

One cross-national study contained 16 countries: Austria, Australia, Belgium, Canada, Denmark, Finland, France, Hungary, Israel, Netherlands, New Zealand, Norway, Sweden, Switzerland, United States of America, and West Germany [1]. Applying the research methods of others [1] to data for 15 of the 16 same countries, the present study examined the relationship between dietary animal fat, beef, pork and alcohol and rates of chronic liver disease and cirrhosis [7]. Hungary had a rate of cirrhosis mortality in 1996 that was so extremely different from what it was in 1965, that it was decided to drop it from the statistical analysis thus leaving only 15 countries instead of 16 countries. The 1996 and 2003 [or closest year(s)] recorded per caput consumption of pure alcohol (liters) per adult 15 years of age and older and the age-standardized rates of mortality (per 100,000 population) were both obtained from the same source, that is, the World Health Organization’s *Global Status Report on Alcohol 1999* and *2004* [9–10]. Online data from the Food and Agriculture Organization of the United Nations [11] provided the per caput supply available for human consumption in kilograms per year for dietary beef (and veal), pork (*i.e.*, pig meat), and animal fats (e.g., butter/ghee, cream, raw animal fats, body oil of fish, liver oil of fish) in 1996 for 15 countries. Data on countries and areas with less than a moderate risk of infection with hepatitis B in 2001 and data on countries where the prevalence of infection with hepatitis C in 2001 was 1–2.5% were both obtained from the same source, *i.e.*, the World Health Organization [12]. For the 1996 (or the closest year) time period the standardized rates of cirrhosis mortality for 15 countries ranged from 2.9 to 16.1 per 100,000 and the ethanol consumption ranged from 1.75 to 13.74 l/caput/yr [9,13]. For the 2003 (or the closest year) time period the standardized rates of cirrhosis mortality for 15 countries ranged from 2.4 to 14.95 per 100,000 and the ethanol consumption ranged from 1.99 to 13.54 l/caput/yr [10,14].

When previous researchers [1] restricted their analysis of 16 countries to only those seven countries with a relatively low and narrow range of alcohol consumption (*i.e.*, 7.5–11.0 l/caput/yr) and a wide range of cirrhosis mortality (2–18 deaths/100,000) they found the correlation was not significant (*r* = 0.023). Repeating this method in the present study produced five of the seven countries originally chosen by the previous researchers [1] using 2003 data and six of the seven countries using 1996 data which still fit with their restriction criteria [13]. West German data were not available since the early 1990’s and 1996 and 2003 data for unified Germany exceeded the criteria and was replaced with data from the Netherlands (which did not exceed the criteria) [14]. Likewise, Switzerland data for 2003 exceeded the criteria and was replaced with data from Finland.

As an extension of the methods of other researchers [1] data were located for 57 countries from the same World Health Organization and United Nations sources as cited below. Some countries were eliminated if they were located in tropical regions where cirrhosis can be caused by infectious disease and also if they were known to have moderate or greater risk of infection with hepatitis B or prevalence rates of hepatitis C infection greater than 2.5%; both forms are known to cause chronic liver disease [12]. Data from 34 countries in 1996 (or closest year) remained available for scrutiny, *i.e.*, Albania, Argentina, Australia, Austria, Belgium, Canada, Cuba, Czech Republic, Denmark, Estonia, Finland, France, Germany, Greece, Iceland, Ireland, Israel, Italy, Latvia, Lithuania, Luxembourg, Macedonia, Malta, Netherlands, New Zealand, Norway, Poland, Portugal, Slovakia, Spain, Sweden, Switzerland, United Kingdom, and United States of America. Again data from these same countries in 2003 (or closest year) were studied, with the exception of Luxembourg.

## 3. Results

**Figure 1.**
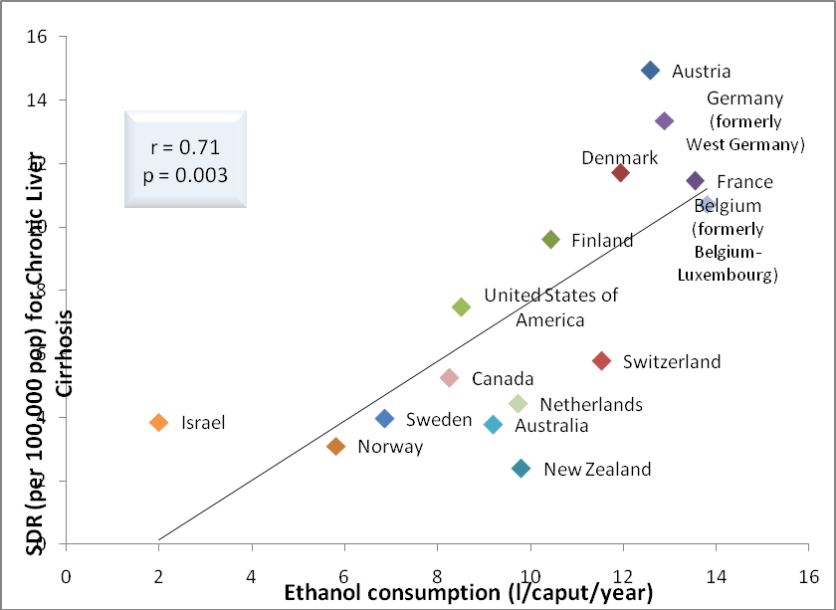
Relationship between cirrhosis mortality and alcohol consumption for 15 countries (2003).

**Figure 2.**
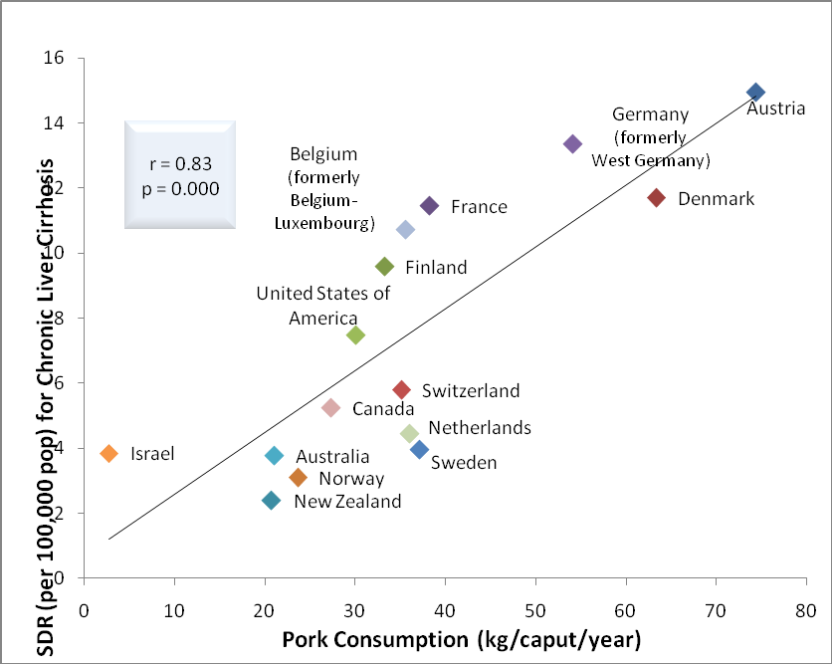
Relationship between cirrhosis mortality and pork consumption for 15 countries (2003).

The correlations between ethanol consumption and rates of cirrhosis mortality were identical, *i.e.*, 0.64 (p = 0.01) in 1996 (or the closest year) and even more significant, *i.e.*, 0.71 (p = 0.003) in 2003 (Figure 1). The 1996 and 2003 correlations of 0.68 (p = 0.005) and 0.83 (p = 0.000), respectively, between dietary pork and rates of cirrhosis mortality (Figure 2). The correlation between dietary animal fat and rates of cirrhosis mortality in 1996 was 0.52 (p = 0.049). In 2003, the correlation between dietary animal fat and rates of cirrhosis mortality was not significant.

In the present study, when correlational analyses were conducted between rates of cirrhosis mortality and the product of dietary fat and alcohol consumption in 1996 and 2003 and were both highly significant, *i.e.*, *r* = 0.70, p =0.004 and *r* = 0.74, p = 0.002, respectively. Similarly, the correlations between rates of cirrhosis mortality and the product of dietary beef and alcohol consumption in 1996 and 2003 were not significant.

The 1996 and 2003 correlations between ethanol consumption and rates of cirrhosis mortality were not significant. Neither were the correlations between dietary beef and dietary fat and rates of cirrhosis mortality for these same years. Using data from seven countries with restriction criteria, the present study’s findings for 1996 and 2003 were not significant.

**Figure 3.**
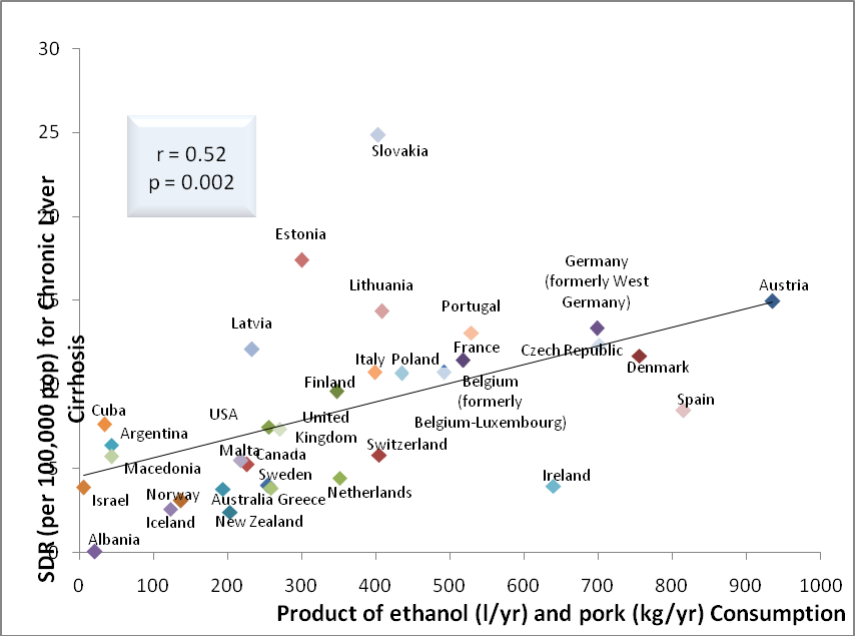
Relationship between cirrhosis mortality and the product of ethanol and pork consumption for 33 countries (2003).

Using 1996 and 2003 data for 34 and 33 countries, respectively, the correlations between ethanol consumption and rates of cirrhosis mortality were 0.55 (p = 0.001) and 0.56 (p = 0.001), respectively, and the correlations between dietary pork and rates of cirrhosis mortality were 0.54 (p = 0.001) and 0.46 (p= 0.008), respectively. In the present study using 2003 data, a significant negative association between dietary beef and rates of cirrhosis mortality was found. Interestingly, the correlations between 1996 rates of cirrhosis mortality and 1) the product of dietary pork and alcohol consumption (Figure 3) and 2) the product of dietary animal fat and alcohol consumption for 34 countries were highly significant (*r* = 0.64, p = 0.000; *r* = 0.45, *p* = 0.008, respectively). Similarly, the correlations between 2003 rates of cirrhosis mortality and 1) the product of dietary pork and alcohol consumption and 2) the product of dietary animal fat and alcohol consumption for 33 countries were also significant (*r* = 0.52, p = 0.002; *r* = 0.52, *p* = 0.002, respectively).

The correlations between cirrhosis mortality and dietary beef, dietary pork, and dietary fat were found not to be significant when restriction criteria were applied, which reduced the sample size to just 14 countries. The present study, using data from this subsample, reports a lack of association between the alcohol consumption and cirrhosis mortality. Similarly, none of the correlations between cirrhosis mortality and the following were significant: 1) ethanol, 2) dietary pork, 3) dietary beef, 4) dietary fat, 5) the products of ethanol and dietary pork, 6) the products of ethanol and dietary beef, and 7) the products of ethanol and dietary fat, using the 14 country subsample.

## 4. Discussion

When data for 15 of the 16 countries used by others [1] were employed, the correlations between ethanol consumption and rates of cirrhosis mortality were identical in 1996 (or the closest year) and even more significant in 2003 (Figure 1). The 1996 and 2003 [7] correlations between dietary pork and rates of cirrhosis mortality were even greater and more significant than that of 0.40 (p < 0.05) reported by other researchers (Figure 2) [1]. The correlation between dietary animal fat and rates of cirrhosis mortality in 1996 was inconsistent with those findings reported by other researchers. That is, no correlation was reported between dietary beef and fat with rates of mortality due to alcoholic cirrhosis [1] and a year later analysis of data from 17 countries indicated that diets high in cholesterol and saturated fat protected (*i.e.*, inversely correlated) against alcoholic cirrhosis while polyunsaturated fats promoted (positively correlated) cirrhosis [8]. The nonsignificant correlation in 2003 between dietary animal fat and rates of cirrhosis mortality is consistent with the results of some [1] but inconsistent with others [8].

Previously, no correlations were reported between the rates of cirrhosis mortality and the product of dietary fat and alcohol consumption and between rates of cirrhosis mortality and the product of dietary beef and alcohol consumption for 16 countries [1]. However, in the present study correlational analyses were conducted between rates of cirrhosis mortality and the product of dietary fat and alcohol consumption in 1996 and 2003 and correlations were highly significant for both years. Previous researchers [1] did not report conducting correlational analyses between rates of cirrhosis mortality and the product of dietary beef and alcohol consumption. However, the present study performed such analyses using data from 1996 and 2003 but the findings were not significant.

Consistent with the results reported by researchers [1] for seven countries, the 1996 and 2003 correlations between ethanol consumption and rates of cirrhosis mortality were not significant. Neither were the correlations between dietary beef and dietary fat and rates of cirrhosis mortality for these same years; however, researchers [1] did not report about either of these two dietary factors correlating with rates of cirrhosis mortality. Finally, researchers [1] reported that the correlation between pork consumption and cirrhosis mortality for the same seven countries was highly significant. The present findings for 1996 and 2003 are inconsistent with their significant correlation using data from seven countries with restriction criteria.

Using 1996 and 2003 data for 34 and 33 countries, respectively, the correlations between ethanol consumption and rates of cirrhosis mortality were both highly significant and the correlations between dietary pork and rates of cirrhosis mortality were also both significant. These findings were consistent with those of other researchers [1] using data from 16 countries; however, the present study used a larger sample of countries more than 30 years later. In contrast to the findings of others [1], the present study using 2003 data reports a significant negative association between dietary beef and rates of cirrhosis mortality.

Interestingly, correlations between deviations from expected cirrhosis mortality and serum uric acid and dietary protein intake suggest that a higher intake of protein may protect against the pathogenesis of liver cirrhosis across countries [8]. The correlations between 1996 rates of cirrhosis mortality and 1) the product of dietary pork and alcohol consumption and 2) the product of dietary animal fat and alcohol consumption for 34 countries were highly significant. Similarly, the correlations between 2003 rates of cirrhosis mortality and 1) the product of dietary pork and alcohol consumption and 2) the product of dietary animal fat and alcohol consumption for 33 countries were also significant. The significant association for rates of cirrhosis mortality and the product of dietary pork and alcohol consumption is supported by others [1] albeit using fewer countries than the present study.

Researchers [1] speculated that pork may be the facilitating factor suggested by other researchers [15] who reported “It appears that the high incidence of cirrhosis among alcoholics is due to a facilitation by alcohol of the effect of some as yet undetermined substance” (p. 68).

When researchers [1] limited alcohol consumption criteria, *i.e.*, (*i.e.*, 7.5–11.0 l/caput/yr) and cirrhosis mortality (2–18 cirrhosis deaths/100,000 population), a subset sample of seven countries was produced. Unlike the only significant association reported by these researchers [1] for dietary pork and cirrhosis mortality, none of the present correlations between cirrhosis mortality and dietary beef, dietary pork, and dietary fat were found to be significant. However, the present lack of an association between the alcohol consumption and cirrhosis mortality was consistent with an earlier seven country sample but not with a mid-1970’s sample of 11 countries using the same restricted alcohol intake and wide range of cirrhosis mortality [1]. When these restriction criteria were applied to the present sample of 34 countries for 1996, only 14 countries remained for statistical analysis. None of the correlations between cirrhosis mortality and the following were significant: 1) ethanol, 2) dietary pork, 3) dietary beef, 4) dietary fat, 5) the products of ethanol and dietary pork, 6) the products of ethanol and dietary beef, and 7) the products of ethanol and dietary fat.

One should be cautious in accepting these results since they may be an artifact of the limitations in the research methods. In the ecologic (population-level) study design, the unit of observation and analysis is a group of individuals rather than individual persons [16]. The ecological study is only able to directly assess group-level associations producing ecological effects. For some researchers this may be the primary focus or goal, however, for others the real focus or goal may be the assessment of individual-level effects [17]. When individual-level associations are already known, ecological effects can be really important from a public health impact point of view [18].

Ecological studies like the present study are susceptible to several types of bias with regard to individual-level associations. This may lead to one of the two types of cross-level inference, *i.e.*, ecological fallacy [17,19]. The fallacy occurs when inferences about individual-level variables, e.g., behavior, are drawn from data about aggregates, e.g., a group of countries [19]. So no attempts should be made to draw inferences regarding individual-level associations in research using an ecological study design [18,19]. In addition, ecological associations may be spurious while the individual associations are not because existing data sources may contain errors, and there is the problem of confounding [17,18]. “Despite these issues, when measurement, analysis, and interpretation are all at the group level and the data sources are reliable, the problems with the ecological approach are minimized” [18, p. 3]. Finally, the caveat that “correlation does not imply causation” applies to the results of the present study.

In the future, mixed or multilevel designs may prove useful as alternatives for the problem of ecological fallacy. These designs are constructed such that the units of comparison include both group-level and individual-level data [18]. Individual-level study results are mixed when it comes to prior investigations of the relationship between type of meat consumed and alcohol consumption and their effects on one’s death from cirrhosis [2,3]. Subsequent research might attempt to clarify the inconsistencies among extant results but on an intra-national level and explain how all of this relates to the individual-level of analysis.

## 5. Conclusions

In summary, the lack of association between alcoholic cirrhosis mortality in 1996 and dietary beef implies that dietary beef may not be factor exacerbating the pathogenesis of alcoholic cirrhosis. Interestingly, the negative correlation between alcoholic cirrhosis mortality in 2003 and dietary beef implies that dietary beef may be protective factor regarding the pathogenesis of alcoholic cirrhosis.

What really stands out in the present study is that regardless the sample of countries, *i.e.*, 15 or 34 (1996) or 33 (2003), the correlations between rates of alcoholic cirrhosis mortality and 1) the product of dietary pork and alcohol consumption and 2) the product of dietary animal fat and alcohol consumption remain significant. However, “none” of these correlations between alcoholic cirrhosis and 1) dietary pork and 2) dietary fat imply a “causal” relationship, although these issues should be investigated further. One should be cautious in accepting these results since they may be an artifact of the limitations associated with using an ecological study design. Another limitation of the present study is the assumption of the immediate effect that pork has on the pathogenesis of cirrhosis. Researchers [1] indicated that data for many countries used in their samples had shown no significant change in pork consumption patterns over the previous decade, *i.e.*, 1965, mid-1970’s, and 1978. Finally, these associations presented may represent a relationship between the risk of alcoholic cirrhosis and some heretofore unknown dietary or environmental factor related to conditions of pork or fat consumption [6,15].
